# Human but Not Mouse Hepatocytes Respond to Interferon-Lambda *In Vivo*

**DOI:** 10.1371/journal.pone.0087906

**Published:** 2014-01-31

**Authors:** Pascale Hermant, Céline Demarez, Tanel Mahlakõiv, Peter Staeheli, Philip Meuleman, Thomas Michiels

**Affiliations:** 1 de Duve Institute, Université Catholique de Louvain, Brussels, Belgium; 2 Institute for Virology, University Medical Center Freiburg, Freiburg, Germany; 3 Spemann Graduate School of Biology and Medicine (SGBM), University Medical Center Freiburg, Freiburg, Germany; 4 Center for Vaccinology, Department of Clinical Chemistry, Microbiology and Immunology, Ghent University and Hospital, Ghent, Belgium; Kantonal Hospital St. Gallen, Switzerland

## Abstract

The type III interferon (IFN) receptor is preferentially expressed by epithelial cells. It is made of two subunits: IFNLR1, which is specific to IFN-lambda (IFN-λ) and IL10RB, which is shared by other cytokine receptors. Human hepatocytes express IFNLR1 and respond to IFN-λ. In contrast, the IFN-λ response of the mouse liver is very weak and IFNLR1 expression is hardly detectable in this organ. Here we investigated the IFN-λ response at the cellular level in the mouse liver and we tested whether human and mouse hepatocytes truly differ in responsiveness to IFN-λ. When monitoring expression of the IFN-responsive Mx genes by immunohistofluorescence, we observed that the IFN-λ response in mouse livers was restricted to cholangiocytes, which form the bile ducts, and that mouse hepatocytes were indeed not responsive to IFN-λ. The lack of mouse hepatocyte response to IFN-λ was observed in different experimental settings, including the infection with a hepatotropic strain of influenza A virus which triggered a strong local production of IFN-λ. With the help of chimeric mice containing transplanted human hepatocytes, we show that hepatocytes of human origin readily responded to IFN-λ in a murine environment. Thus, our data suggest that human but not mouse hepatocytes are responsive to IFN-λ *in vivo*. The non-responsiveness is an intrinsic property of mouse hepatocytes and is not due to the mouse liver micro-environment.

## Introduction

The type III interferon (IFN) family was discovered about 10 years ago by two independent groups [1], [2]. Three type III IFN subtypes have been described, IFN-λ1, IFN-λ2 and IFN-λ3, also named IL29, IL28A and IL28B, respectively [1], [2]. A fourth IFN-λ-coding sequence was found in some allelic variants [3]. In mice, only IFN-λ2 and IFN-λ3 are expressed since IFN-λ1 is a pseudogene [1], [2], [4]. Type III IFNs signal through a heterodimeric receptor composed of the IFN-λ-specific IFNLR1 chain and the IL10RB chain which is shared by other IL-10 cytokine family members [1], [2], [5]. Although type I and type III IFNs use distinct receptors, they both signal via the Jak-STAT pathway, leading to phosphorylation of STAT1 and STAT2 and to formation of the ISGF3 complex by association with IRF9. Consequently, type I and type III IFNs upregulate the same group of IFN-stimulated genes (ISGs), which code for proteins such as Mx or OAS that mediate antiviral activity [4]–[7]. However, type I and type III IFNs differ with regard to their receptor distribution. While the type I IFN receptor (IFNAR) is reportedly expressed by all nucleated cells, the IFNLR1 chain of the type III IFN receptor is preferentially expressed by epithelial cells [8]. Consequently, IFN-λ is expected to be a good alternative to type I IFNs for the treatment of some viral diseases, as fewer side effects are expected due to the more restricted range of IFN-λ target cells. Accordingly, IFN-λ entered phase III clinical trials as a candidate drug against hepatitis C virus (HCV) infection.

Due to their epithelial nature, hepatocytes are expected to respond to IFN-λ. Studies with human primary hepatocytes convincingly showed that these cells express the IFN-λ receptor complex and respond to IFN-λ [9]–[11]. Further, IFNLR1 was shown to be expressed in primary human hepatocytes and in some human hepatoma cell lines. Accordingly, Mx and OAS were induced after treatment of primary human hepatocytes with pegylated IFN-λ1 [11]. More recently, Dickensheets *et al.* confirmed responsiveness of primary human hepatocytes, although the magnitude of ISG expression in response to IFN-λ was lower than that induced by an equivalent concentration of IFN-α [9]. Muir *et al.* observed an antiviral activity of IFN-λ against HCV in chronically infected patients [12]. In contrast, in the mouse, the IFN-λ response is very weak in the liver and IFNLR1 expression is hardly detected in this organ [8], [13]. Accordingly, IFN-λ was ineffective against hepatotropic viruses such as Thogoto virus and Rift valley virus [13]. Further, in mice expressing the firefly luciferase gene under transcriptional control of the IFN-inducible *Mx2* promoter, injection of IFN-λ did not induce a detectable response in the liver [14]. Consistent with these data, Pagliacetti and colleagues found that IFN-λ2 only weakly inhibited HBV replication when injected intravenously in HBV-transgenic mice, as compared to the inhibition observed with IFN-β or IFN-γ [15].

As a first approach to more thoroughly investigate the IFN-λ response in the mouse liver, we used mice carrying a functional *Mx1* gene [16] that permit measuring IFN responses with high sensitivity at the single cell level [8], [17]. Using this system, we found that although mouse hepatocytes can respond to IFN-α, they do not respond to IFN-λ. Instead, we observed a strong IFN-λ response in cholangiocytes, the epithelial cells forming the bile ducts. Finally, using chimeric mice that were transplanted with human hepatocytes [18], we demonstrated that human but not mouse hepatocytes are responsive to IFN-λ under identical experimental conditions *in vivo*.

## Materials and Methods

### Animal Experiments

Ethics statement: Handling of mice (agreement LA1230472) and experimental procedures were conducted in accordance with the EEC directive 86/609/CEE and the related Belgian law of April 6th 2010. The study and protocol used in this study were approved by the ethics committee of the University of Louvain under the agreement # 2010/UCL/MD/031.

Six to 9 week-old C57BL/6 mice carrying a functional *Mx1* gene (B6.A2G-*Mx1*, designated Mx-wt) were from the breeding colony at the University of Freiburg, Germany. B6.A2G-*Mx1* mice either lacking a functional type I IFN receptor (designated Mx-IFNAR1^0/0^), a functional type III IFN receptor (designated Mx-IFNLR1^0/0^) or both (designated Mx-IFNAR1^0/0^ IFNLR1^0/0^) were described previously [13]. 12 to 13 week-old chimeric human liver-uPA-SCID mice were generated in the animal facility of the Faculty of Medicine and Health Sciences at the Ghent University. Briefly, 2-week old transgenic SCID mice overexpressing the urokinase-type plasminogen activator (uPA) gene under the control of the albumin promoter were xenografted with about 1 million primary human hepatocytes (donor HH223; BD Biosciences, Erembodegen, Belgium) as previously described [18]. Engraftment and repopulation of the mouse liver by the transplanted human hepatocytes was evaluated by quantifying the amount of human albumin present in the mouse plasma (Bethyl Laboratories, Montgomery, Tx).

### Plasmids

All plasmids used in this work are pcDNA3 (Invitrogen) derivatives. The primer sequences used in this work are presented in table 1. The plasmids pcDNA3-muIFNαA, pcDNA3-muIFNα6T and pcDNA3-muIFNλ3 were described previously [8], [19]. HuIFN-λ3 was cloned from TOPO-huIFNλ3 (Source Bioscience) into pcDNA3 (Invitrogen). The PCR-amplified fragment was cloned into pcDNA3 using the HindIII and Asp718 restriction sites. HuIFN-α2 was amplified from HeLa cells DNA, The PCR-amplified fragment was cloned into pcDNA3 using *Eco*RI and *Not*I restriction sites.

**Table 1:** Primers.

Gene amplified	Primer sequencec,d
huIFN-λ3	(f) 5′-AAA AAA GCT TAC CAT GAC CGG GGA CTG CAT GCC-3′
	(r) 5′-AAA AGG TAC CTC AGA CAC ACA GGT CCC CGC T-3′
huIFN-α2	(f) 5′-AAA AGA ATT CAC CAT GGC CTT GAC CTT TGC TTT-3′
	(r) 5′-AAA AGC GGC CGC TCA TTC CTT ACT TCT TAA ACT TT-3′
influenza A	(f) 5′-AAG ACC AAT CCT GTC ACC TCT GA-3′
	(r) 5′-CAA AGC GTC TAC GCT GCA GTC C-3′
mOASl2	(f) 5′-GGA TGC CTG GGA GAG AAT CG-3′
	(r) 5′-TCG CCT GCT CTT CGA AAC TG-3′
hOAS1^a^	(f) 5′-AGA AAG AGG GCG AGT TCT CC-3′
	(r) 5′-TGG GCT GTG TTG AAA TGT GT-3′
hMxA^b^	(f) 5′-TTC AGC ACC TGA TGG CCT ATC-3′
	(r) 5′-CCG TAC GTC TGG AGC ATG AAG-3′
β-actin	(f) 5′- AGA GGG AAA TCG TGC GTG AC-3′
	(r) 5′- CAA TAG TGA TGA CCT GGC CGT-3′

a Sequence kindly provided by Professor Stephan Brand, University Hospital Munich-Grosshadern, University of Munich, Germany.

b [32].

c (f) forward primer; (r) reverse primer.

d For RT-qPCR, annealing reactions were performed at 63°C for influenza A virus and at 60°C for mOASl2, hOAS1, hMxA and β-actin.

### Virus Infection

TURH (H7/N1) virus is a mouse-adapted hepatotropic variant of influenza virus strain A/TURKEY/England 63 (Havl, Nav3) [20]. 8–9 week-old Mx-IFNAR1^0/0^ and Mx-IFNLR1^0/0^ mice were used in this experiment. For each genotype, twelve mice were infected intraperitoneally with 10^4^ plaque-forming units (pfu) of TURH. Livers were collected at 24 h (n = 2) and 48 h (n = 6) post infection. Four mice, used as negative control, received phosphate buffer saline (PBS) and were sacrificed after 48 h.

### DNA Electroinjection and Recombinant IFN Administration

Mx-wt mice or chimeric human liver uPA-SCID mice were anesthetized and electroinjected as previously described [8]. Briefly, 10 µg of endotoxin-free plasmid DNA (Quiagen endofree) suspended in PBS, were injected in a volume of 25 µl in the left and right tibialis anterior muscles. DNA was electroporated using a Cliniporator system (Cliniporator, IGEA, Carpi, Italy). Mice were then woken up by intraperitoneal injection of 250 µl of Atipamezol 500 µg/ml (Antisedan) and kept in an insulator for 7 days prior to sacrifice and organ harvest. For recombinant IFN administration, 6–8 week-old Mx-IFNAR1^0/0^, Mx-IFNLR1^0/0^ or Mx-IFNAR1^0/0^ IFNLR1^0/0^ were treated subcutanously with 100 µl of PBS containing either 1 µg of cross-reactive human IFN-αB/D [21], 1 µg of recombinant mouse IFN-λ2 (PeproTech), or a mixture of both cytokines.

### RNA Extraction and Quantitative Real-time Reverse Transcription (RT-qPCR)

Mice were anesthetized and perfused intracardially with PBS before organs harvest. RNA was isolated from organs, reverse-transcribed and subjected to quantitative RT-PCR (RT-qPCR) as previously described [22], using SybrGreen and the MyIQ™ apparatus (Biorad). The primer sequences used are described in Table 1. Standards consisted of 10-fold dilutions of known concentrations of a plasmid carrying a PCR fragment from cDNA encoding the influenza A virus matrix protein M1, OASl2, OAS1, MxA or β-actin. All RT-qPCR results were normalized to β-actin cDNA levels in a sample.

### Immunohistofluorescence

Mx-wt mice or chimeric human liver-uPA-SCID mice were anesthetized and perfused intracardially with PBS containing 1–4% of paraformaldehyde (PFA) before organ harvest. Freshly collected organs were immersed in 4% buffered formaldehyde for 4 h at room temperature and then paraffin-embedded. Tissues were cut at 4 µm with a microtome and sections were placed on SuperFrost Plus slides, dried at 37°C overnight, and processed by standard methods for immunohistochemistry. Briefly, tissue sections were deparaffinized, permeabilized for 5 min in PBS/0.1% Triton X-100 and washed in PBS. Unmasking of antigens was done by treating tissue sections for 60 min at 97°C in 10 mM sodium citrate buffer (pH 5.8) before blocking with Antibody Diluent with Background Reducing Components (Dako #S3022). Primary and secondary antibodies were diluted in blocking solution.

The following primary antibodies and dilutions were used: rabbit polyclonal anti-influenza virus (FLUAV #178, Freiburg University) at 1∶1000, rabbit polyclonal anti-mouse Mx1 at 1/200 (AP5#2771, [23]), mouse monoclonal anti-human HNF4α/NR2A1 (HNF4) at 1/300 (R&D Systems #PP-H1415-00), mouse monoclonal anti-pan cytokeratin at 1/500 (Sigma, #C5992), mouse monoclonal anti-human MxA at 1/2500 (#M143 [24]), rabbit polyclonal anti-human MxA at 1/2500 (#41, Freiburg University), mouse monoclonal anti-human cytokeratin 18 (Clone DC 10, Dako, # M 7010), rabbit polyclonal anti-human albumin (Merck Millipore, #126584).

Secondary antibodies were AlexaFluor conjugates (Invitrogen). The following antibodies and dilutions were used: goat anti-mouse AlexaFluor 594 cross-adsorbed at 1/400 (HNF4 and Cytokeratin immunostainings), goat anti-rabbit AlexaFluor 594 at 1/400 (albumin immunostainings), goat anti-rabbit HRP at 1/100 (influenza virus, Mx1 and MxA (#41) immunostainings) and goat anti-mouse HRP at 1/100 (MxA (#143) immunostainings). For influenza A virus, Mx1 and MxA immunostainings, detection required amplification using tyramide signal amplification (TSA) (#NEL701001KT and NEL702001KT, PerkinElmer). Nuclei were stained with Hoechst33258 (Roche) or DAPI (Vectashield H-1200, Vector Laboratories).

### Statistical Analysis

Data were analyzed with Prism version 4.0c using one-tailed Mann-Whitney *U* test. *P* values ≤0.05 were considered significant.

## Results

### Cholangiocytes but not Hepatocytes Respond to IFN-λ in Mouse Livers

**Figure 1:**
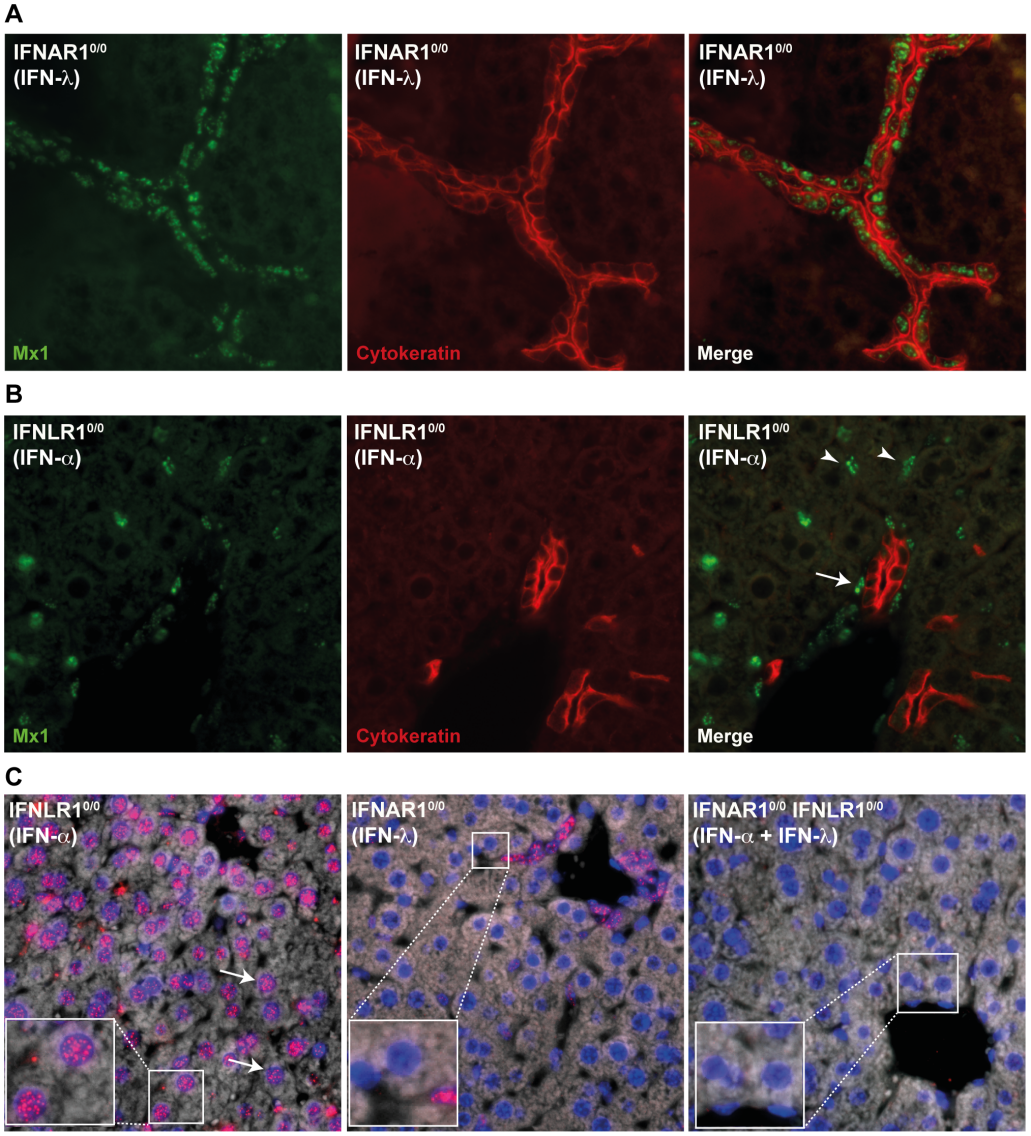
Cholangiocytes are the main IFN-λ-responsive cells in mouse livers. **A:** Fluorescence microscopy images showing co-immunostainings of Mx1 (green) and cytokeratin (red) in liver sections from Mx-IFNAR1^0/0^ mice electroinjected with IFN-λ expression plasmid. **B:** Fluorescence microscopy images showing co-immunostainings of Mx1 (green) and cytokeratin (red) in liver sections from Mx-IFNLR1^0/0^ mice electroinjected with IFN-α expression plasmid. Arrowheads point to intersticial cells; arrow points to endothelial cell. **C:** Immunofluorescent detection of Mx1 (red) in liver sections of mice that received a single subcutaneous dose of IFN. Left panel: Mx-IFNLR1^0/0^ mice inoculated with IFN-α; central panel: Mx-IFNAR1^0/0^ mice inoculated with IFN-λ; right panel: control Mx-IFNAR1^0/0^ IFNLR1^0/0^ inoculated with IFN-α and IFN-λ. Nuclei were stained with DAPI. Arrows point to Mx1-positive hepatocytes identified by their large nuclei.

Previously published data from *in vivo* experiments show a very weak IFN-λ response in the mouse liver [8], [13]. As a first approach to identify IFN-λ-responsive cells in mouse liver, we performed Mx1 immunolabelings on liver sections from mice that were electroinjected with IFN expression vectors. We used the previously described mice that carry a functional *Mx1* gene so that the IFN response can be detected by immunohistofluorescence using antibodies detecting Mx1, which gives a characteristic nuclear signal. Mx-IFNLR1^0/0^, Mx-IFNAR1^0/0^ or Mx-IFNAR1^0/0^ IFNLR1^0/0^ mice were electroinjected in the tibialis anterior muscle with IFN-λ3 or IFN-α6T expression vectors, or with the empty vector (pcDNA3). This method allows a long lasting expression of circulating IFN [8]. Mx1 immunodetection was used to identify IFN-responding cells. IFN-λ-responding cells were identified in Mx-IFNAR1^0/0^ mice electroinjected with the IFN-λ-expressing plasmid, whereas IFN-α-responding cells were identified in Mx-IFNLR1^0/0^ mice electroinjected with the IFN-α-expressing plasmid. Mx-IFNAR1^0/0^ IFNLR1^0/0^ mice served as negative controls. We observed a response to IFN-λ in cytokeratin-positive cholangiocytes, but not in hepatocytes (Fig. 1A). The response to IFN-α differed strikingly from the response to IFN-λ. Many interstitial cells as well as endothelial cells responded to IFN-α. Surprisingly, however, no IFN-α response was observed in hepatocytes (Fig. 1B). As expected, the liver of Mx-IFNAR1^0/0^ IFNLR1^0/0^ mice responded neither to IFN-α nor to IFN-λ, although very few unidentified interstitial cells appeared to constitutively express Mx1 (data not shown).

It was reported that prolonged stimulation of liver cells with IFN-α triggers desensitization of the IFN response pathway [25]. Such refractoriness was not observed, however, in the case of IFN-λ [26]. To rule out that the lack of Mx1 expression in mouse hepatocytes was related to IFN refractoriness, we monitored Mx1 expression in the liver of mice subcutaneously injected with a single dose of recombinant IFN-α or IFN-λ (Fig. 1C). In this case, Mx1 expression could be readily detected in hepatocytes of Mx-IFNLR1^0/0^ mice treated with IFN-α. However, as in the previous experiment, no Mx1 expression was detected in hepatocytes of Mx-IFNAR1^0/0^ mice treated with IFN-λ.

### Hepatocytes do not Respond to IFN-λ in Livers Infected with a Hepatotropic Strain of Influenza A Virus

**Figure 2:**
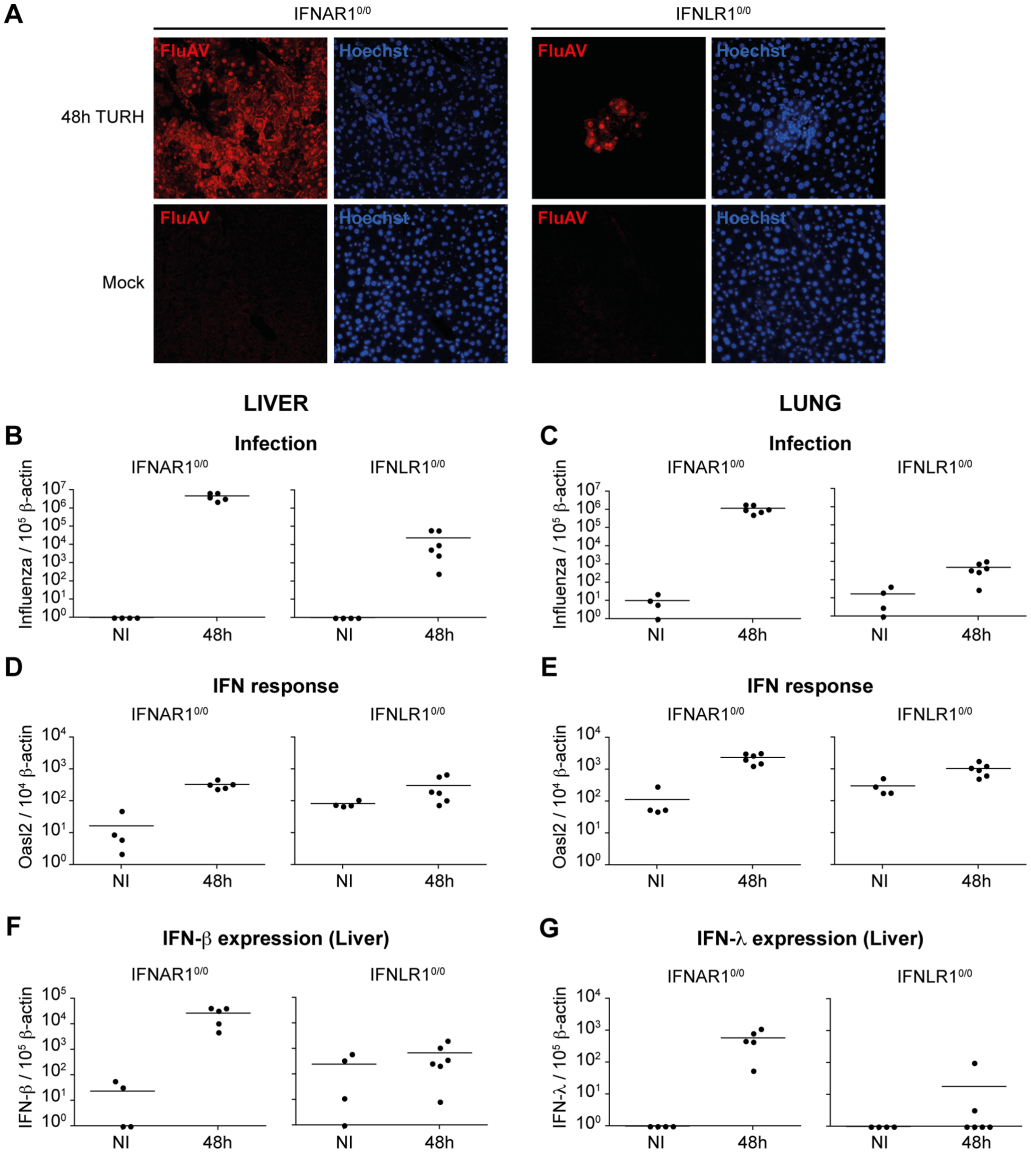
IFN response in mice infected with the TURH influenza A virus strain. **A:** Immunofluorescence detection of TURH influenza A virus in liver sections from Mx-IFNAR1^0/0^ and Mx-IFNLR1^0/0^ mice infected for 48 hours or mock-infected, using anti-influenza A virus antibody (Red) and Hoechst staining of nuclei (blue). Results are representative of two independent experiments. **B–C:** RT-qPCR analysis of influenza A virus replication in the liver (B) and lungs (C) of Mx-IFNAR1^0/0^ and Mx-IFNLR1^0/0^ mice. Results are expressed as influenza A virus cDNA copies per 10^5^ copies of β-actin cDNA. **D–E:** RT-qPCR analysis of *Oasl2* expression in the liver (D) and lungs (E) of Mx-IFNAR1^0/0^ and Mx-IFNLR1^0/0^ infected mice. Results are expressed as *mOasl2* cDNA copies per 10^3^ copies of β-actin cDNA. **F–G:** IFN-β (F) and IFN-λ (G) production in the liver of infected mice. Results are expressed as *IFN* cDNA copies per 10^5^ copies of β-actin cDNA. It is noteworthy that cells from IFNAR1^0/0^ expressed lower basal levels of IFN, likely as a consequence of a disrupted positive feed-back loop for IFN expression in these mice.

The apparent absence of IFN response in hepatocytes observed in the previous experiment was unexpected. It might be explained by low-level supply of IFN from the bloodstream after plasmid electroinjection-based expression in mice. This artificial system might not mimic the local high concentrations of IFN which are probably reached in virus-infected tissues. To test whether hepatocytes can respond to high local concentrations of IFN, Mx-IFNLR1^0/0^ and Mx-IFNAR1^0/0^ mice were infected for 24 (not shown) and 48 hours, respectively, with mouse hepatotropic influenza A virus strain TURH. RT-qPCR and immunohistochemistry confirmed the infection of livers and lungs (Fig. 2A–C). Livers and lungs of Mx-IFNAR1^0/0^ mice were much more strongly infected than those of Mx-IFNLR1^0/0^ mice, in agreement with the previous report showing stronger influence of type-I IFN on influenza A virus infection [13]. Both in the liver and in the lung, *Oasl2* mRNA expression was strongly induced in IFNAR1^0/0^ mice (Fig. 2D–E), suggesting that these tissues readily responded to IFN-λ produced in response to infection. IFN was produced locally in response to viral infection, as transcription of both IFN-β and IFN-λ genes was strongly upregulated in the livers of infected Mx-IFNAR1^0/0^ mice (Fig. 2F–G).

**Figure 3:**
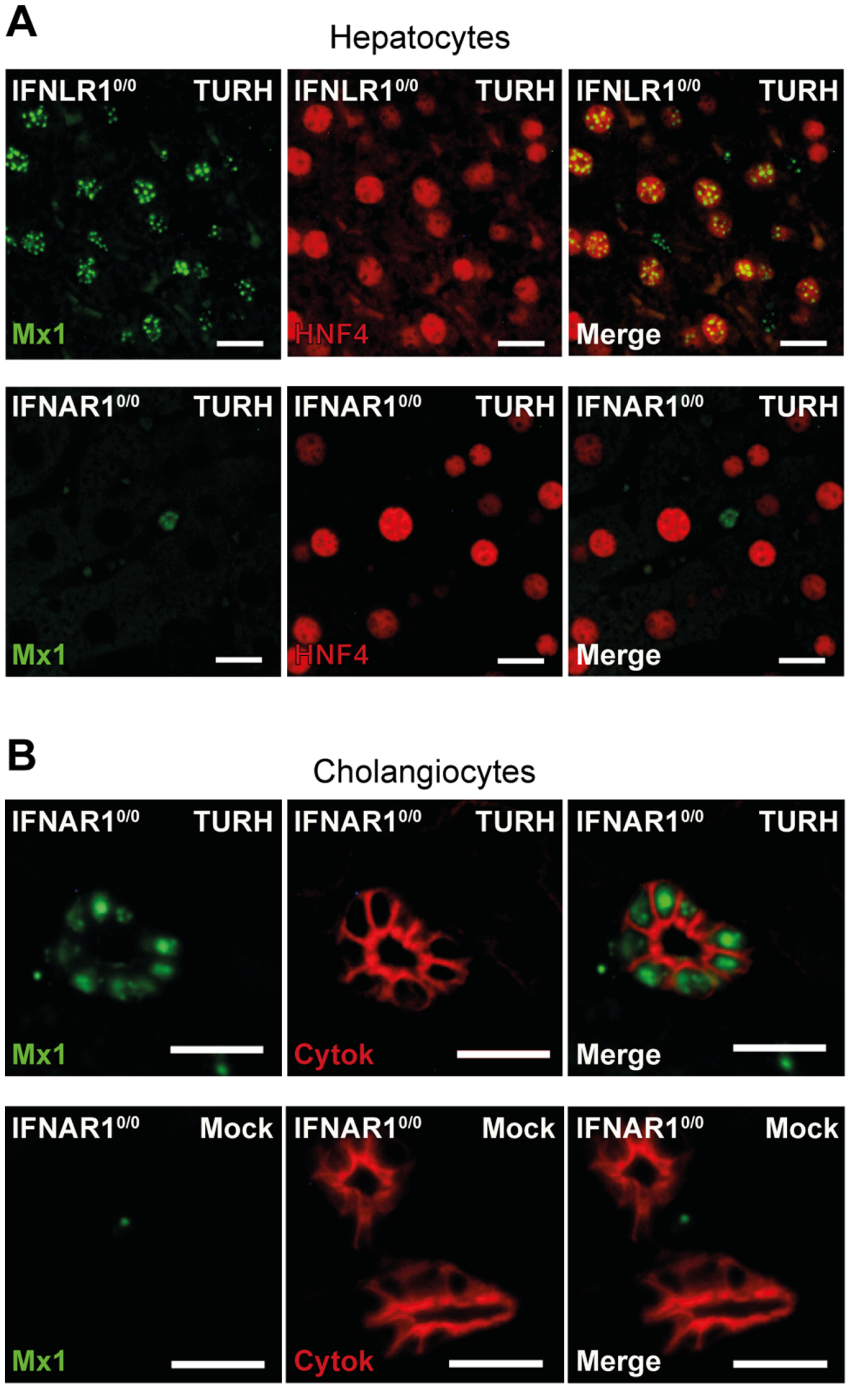
Hepatocytes respond to IFN-α but not to IFN-λ. **A:** Fluorescence microscopy images showing the IFN response (Mx1, green) in hepatocytes (HNF4, red), in liver sections from Mx-IFNLR1^0/0^ and Mx-IFNAR1^0/0^ mice infected for 48 hours with influenza A virus strain TURH. Scale bar: 15 µm. **B:** Fluorescence microscopy images showing the IFN-λ response (Mx1, green) in cholangiocytes (cytokeratin, red) in liver sections from Mx-IFNAR1^0/0^ mice, mock-infected or infected for 48 hours with the TURH strain of influenza A virus or mock-infected. Scale bar: 15 µm. Results are representative of two independent experiments.

To study the IFN response in hepatocytes, we performed Mx1 immunolabelings on liver sections from mice infected for 48 hours (Fig. 3). In infected Mx-IFNLR1^0/0^ mice, many scattered Mx1-positive hepatocytes were detected in the liver (Fig. 3A), with the response being most intense close to infection foci, confirming the ability of hepatocytes to respond to type I IFN. In contrast, no response to type III IFN was detected in hepatocytes of Mx-IFNAR1^0/0^ mice. In livers of these mice, the only cells responding to type III IFN were the cholangiocytes, as previously observed with electroinjected animals (Fig. 3B). Thus, in spite of massive local IFN production, hepatocytes did not respond to type III IFN, while they responded to type I IFN.

### Human but not Mouse Hepatocytes Respond to IFN-λ

The data presented above, as well as previously published works show that, unlike human hepatocytes [9], [11], mouse hepatocytes do not respond to IFN-λ [8], [13]. This raised the question of whether the lack of responsiveness of mouse hepatocytes to IFN-λ was due to the nature of the hepatocytes, or to the micro-environment in mouse livers. To answer this question we used Alb-uPA-SCID transgenic mice, which were engrafted with human hepatocytes. These mice have chimeric livers containing both mouse and human hepatocytes [18], [27], [28].

**Figure 4:**
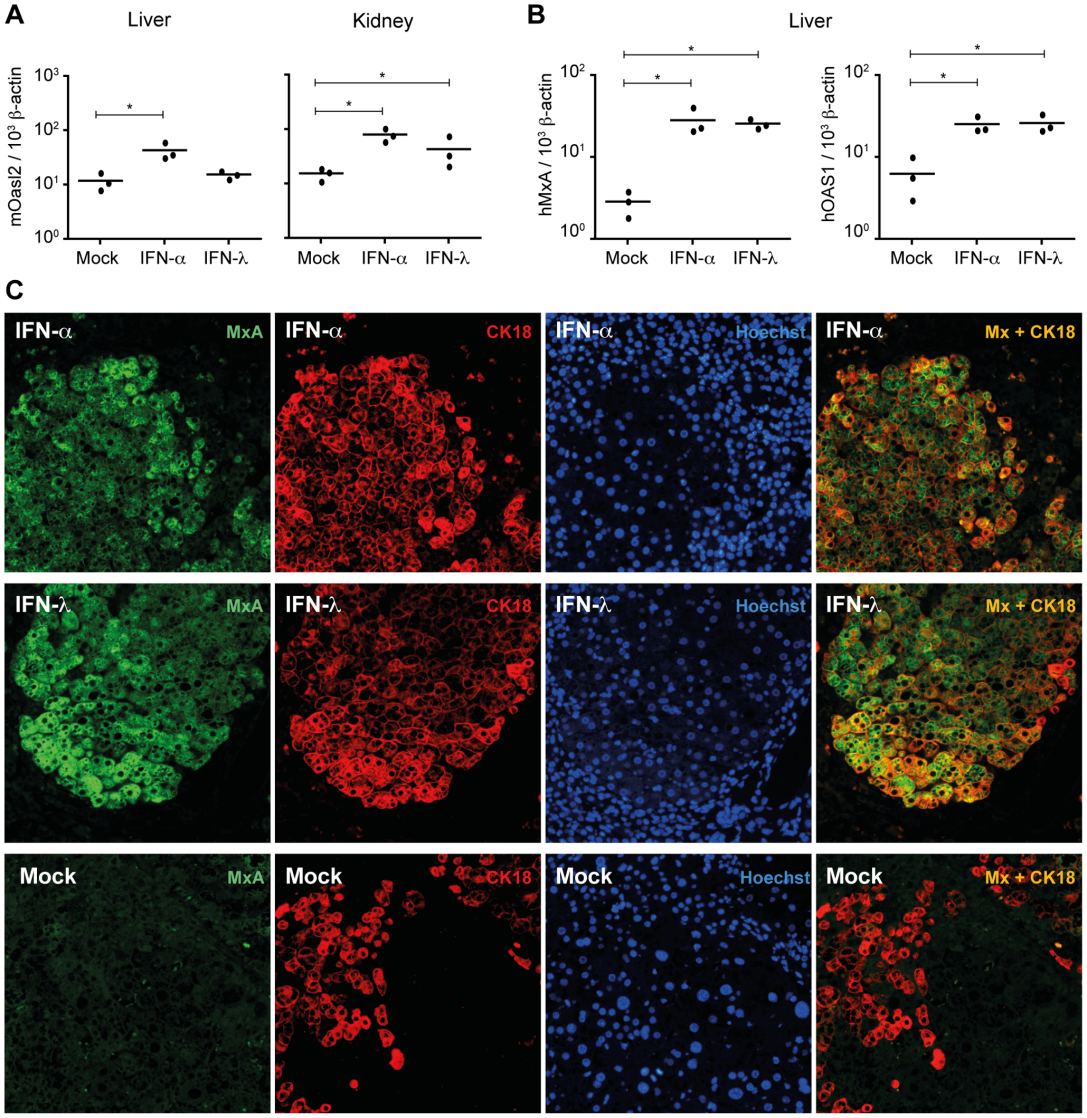
Human hepatocytes in a mouse model respond to IFN-λ. **A:** RT-qPCR analysis of mouse *Oasl2* (*mOasl2*) expression in the livers and kidneys of Alb-uPA-SCID transgenic mice electroinjected with IFN-α or IFN-λ expression plasmids or with the empty plasmid (mock). Results are expressed as *mOasl2* cDNA copies per 10^3^ copies of β-actin cDNA **B:** RT-qPCR analysis of human *MxA* (*hMxA*) and *OAS1* (*hOAS1*) expression in the livers of Alb-uPA-SCID transgenic mice electroinjected with IFN-α or IFN-λ expression plasmids or with the empty plasmid (mock). Results are expressed as *hMxA* or *hOAS1* cDNA copies per 10^3^ copies of β-actin cDNA. *p≤0.05. **C:** Fluorescence microscopy images showing co-immunostainings of human MxA and human cytokeratin 18 in liver sections of Alb-uPA-SCID transgenic mice electroinjected with IFN-α or IFN-λ expression plasmids or with the empty plasmid (mock). Hoechst: nuclear staining. Results are representative of two independent experiments.

Chimeric mice were electroinjected either with a plasmid expressing human IFN-λ3, or a mix of plasmids expressing human and mouse IFN-α, or with the empty vector (pcDNA3). The antiviral activity of type I IFN is species-specific [29], [30]. Therefore, in order to monitor the IFN-α response in chimeric livers, a 50∶50 mixture of plasmids expressing human IFNα2 and mouse IFN-αA was used for electroinjection. In contrast, mouse cells can respond to human IFN-λ and *vice versa* (Fig. S1). Therefore, we used a single plasmid expressing hIFN-λ3 for electroinjection. At 7 days post-electrotransfer, ISG expression was measured in the kidneys and livers, using RT-qPCR, to evaluate the overall IFN response (Fig. 4A–B). The response of mouse cells was quantified by measuring expression of mouse *Oasl2*. The response of human hepatocytes was quantified by measuring expression of human *OAS1* and *MXA*. In IFN-α-expressing mice, we observed a significant increase of mouse *Oasl2* transcripts in chimeric livers (p = 0.05). As in previous experiments, no significant mouse *Oasl2* response to IFN-λ was detected in livers (p = 0.2), while a clear response to this cytokine was observed in kidneys (p = 0.05) (Fig. 4A). Importantly, we observed very good human *OAS1* and *MXA* responses to IFN-λ in chimeric livers (p = 0.05) (Fig. 4B).

The prominent IFN-λ response of human hepatocytes was further visualized by immunohistofluorescence analysis of liver sections. Human hepatocytes were identified using anti-human cytokeratin 18 (Fig. 4C) or anti-human albumin antibodies (Fig. S2). In chimeric mice treated with either IFN-λ or IFN-α, most human hepatocytes were strongly positive for MxA. Taken together, these results show that human hepatocytes in a mouse environment are able to respond to type III IFN.

## Discussion

In this work, we highlight the lack of responsiveness of mouse hepatocytes to IFN-λ *in vivo*. Even when high amounts of IFN-λ were produced locally after influenza A virus infection, the IFN-λ response was confined to cholangiocytes. Previous works showed that mouse livers responded poorly to IFN-λ [8], [13]. However, using RT-qPCR and Northern blot analysis a recent study showed that primary mouse hepatocytes can respond to IFN-λ [9]. Nonetheless, since these results were obtained *in vitro*, it remains possible that the hepatocytes did not behave exactly as they would *in vivo*. Here, we analyzed the IFN-λ response, at the cellular level, in the intact mouse liver. We show that cholangiocytes were the predominant cells responding to IFN-λ in mouse liver. No response to IFN-λ was observed in hepatocytes, even in infected livers where high amounts of IFN were expected to be produced locally.

Human hepatocytes, however, were shown to be responsive to IFN-λ *in vitro* [9], [11]. *In vivo*, good antiviral activity was observed against HCV with PEG-IFN-λ [12]. Clinical studies are currently ongoing for the use of IFN-λ in the treatment of viral diseases in human.

However, to our knowledge, a formal proof that hepatocytes directly respond to IFN-λ is still lacking. The most compelling evidences that IFN-λ acts on human hepatocytes are the detection of IFNLR1 by immunohistochemistry on hepatocytes in sections of hepatic biopsies [11], and the relatively high expression level of IFNLR1 mRNA in human hepatocytes engrafted in mice with chimeric livers [31].

Using Alb-uPA-SCID mice with chimeric livers we demonstrated that human hepatocytes present in a mouse environment can respond to circulating IFN-λ. This result does not unequivocally prove that human hepatocytes are physiologically responsive to IFN-λ. Indeed, although these hepatocytes were not cultured *in vitro*, one cannot completely rule out the fact that IFNLR1 expression was consequent to the hepatocyte isolation procedure. Yet, it is very likely that human hepatocytes do respond to IFN-λ and we postulate that epigenetic factors might silence IFN-λ receptor expression in mouse but not human hepatocytes.

Our results further show that the lack of responsiveness of mouse hepatocytes to IFN-λ is not due to the murine environment since human hepatocytes present in the same tissue readily responded to IFN-λ.

In conclusion, our data suggest that human but not mouse hepatocytes are responsive to IFN-λ. This highlights the existence of some limits to the use of mouse models in the study of human hepatotropic pathogens.

## Supporting Information

Figure S1 **Human-mouse cross-reactivity of IFN-λ.** A–B. RT-qPCR analysis of muOASl2 in LKR-10 cells (A) and huMxA in HeLa cells (B) treated with human or mouse IFN-α and IFN-λ. Means and SD of 4 samples. *: p≤0.05, NS: non significant.
(TIF)

Figure S2 **Human hepatocytes in a mouse model respond to IFN-λ.** Fluorescence microscopy images showing co-immunostainings of human MxA and human albumin in liver sections of Alb-uPA-SCID transgenic mice electroinjected with IFN-α or IFN-λ expression plasmids or with the empty plasmid (mock). Hoechst: nuclear staining. Results are representative of two independent experiments.
(TIF)

Materials and Methods S1 This section describes the cells [33], [34] and procedures used to produce and quantify IFNs.
(DOCX)
