# Supplementary figures and images for: Human but Not Mouse Hepatocytes Respond to Interferon-Lambda In Vivo
Source: PLoS One. 2014 Jan 31;9(1):e87906. doi: 10.1371/journal.pone.0087906 (PMC3909289; doi:10.1371/journal.pone.0087906)

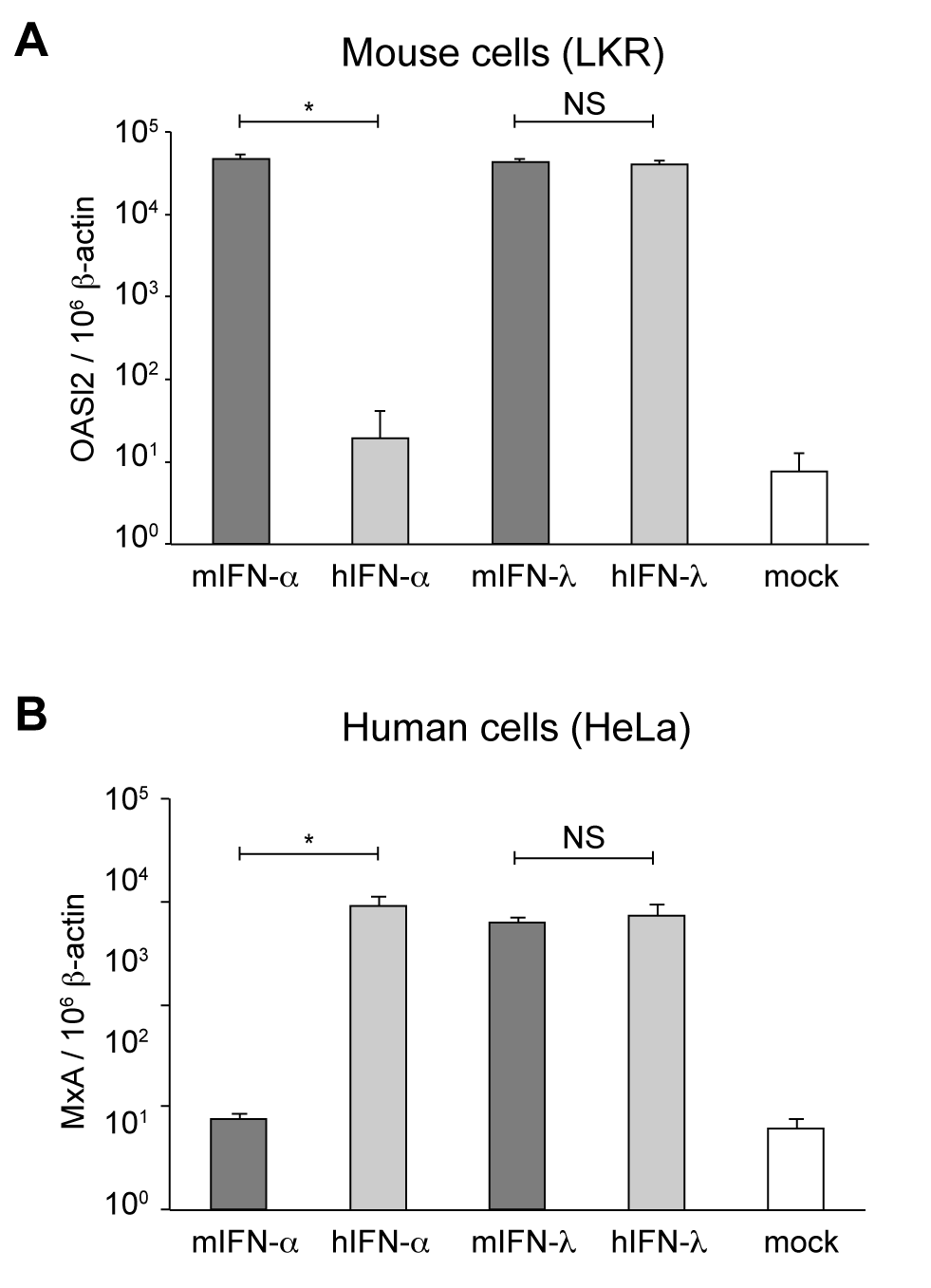

Supplement: Figure S1 — Human-mouse cross-reactivity of IFN-λ. A–B. RT-qPCR analysis of muOASl2 in LKR-10 cells (A) and huMxA in HeLa cells (B) treated with human or mouse IFN-α and IFN-λ. Means and SD of 4 samples. *: p≤0.05, NS: non significant. (TIF) [file pone.0087906.s001.tif]

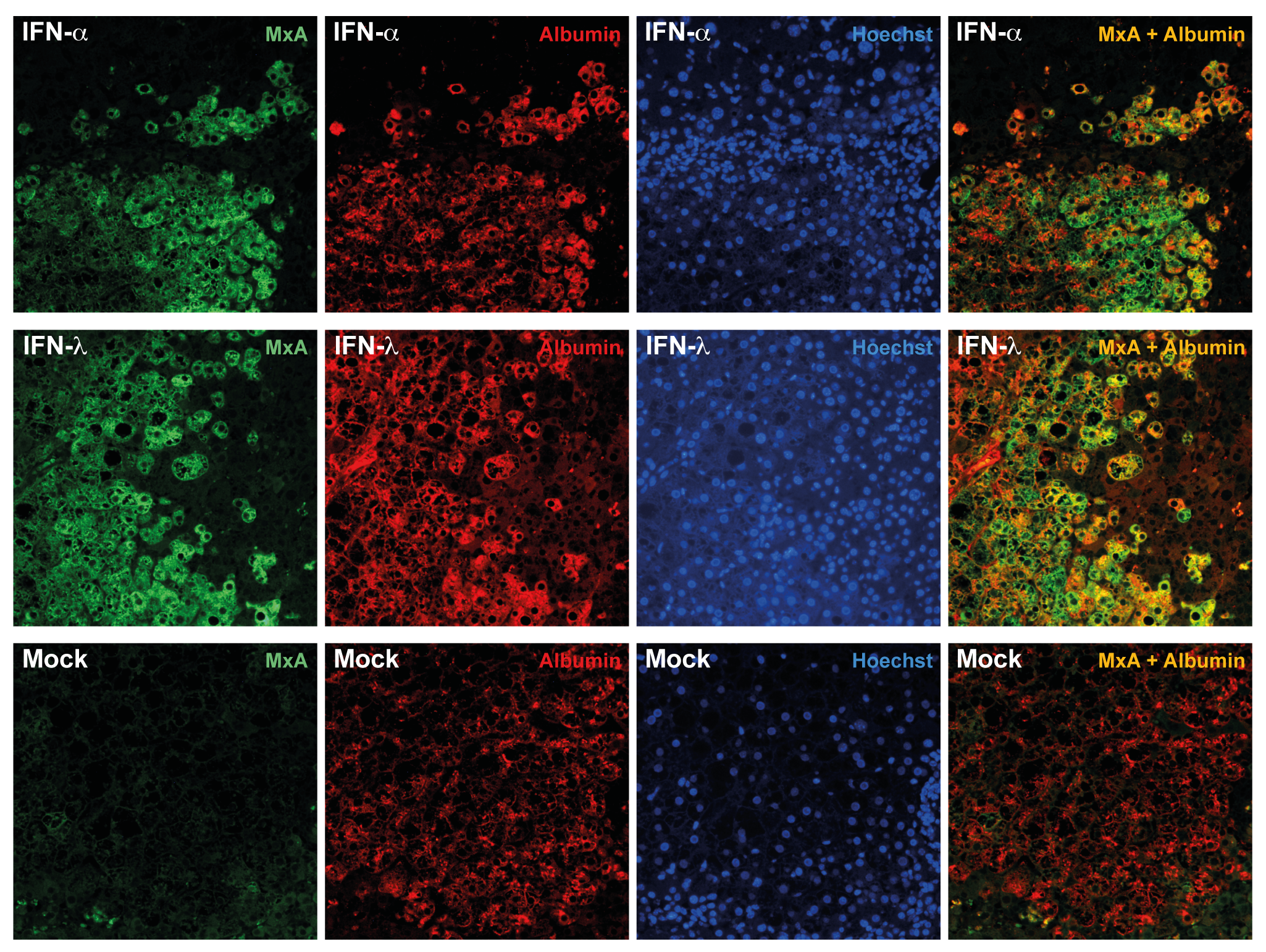

Supplement: Figure S2 — Human hepatocytes in a mouse model respond to IFN-λ. Fluorescence microscopy images showing co-immunostainings of human MxA and human albumin in liver sections of Alb-uPA-SCID transgenic mice electroinjected with IFN-α or IFN-λ expression plasmids or with the empty plasmid (mock). Hoechst: nuclear staining. Results are representative of two independent experiments. (TIF) [file pone.0087906.s002.tif]
