# Supplementary material for: Human but Not Mouse Hepatocytes Respond to Interferon-Lambda In Vivo
Source: PLoS One. 2014 Jan 31;9(1):e87906. doi: 10.1371/journal.pone.0087906 (PMC3909289; doi:10.1371/journal.pone.0087906)
Supplement: Materials and Methods S1 — This section describes the cells [33], [34] and procedures used to produce and quantify IFNs. (DOCX) [file pone.0087906.s003.docx]

**S3. Supporting Materials and Methods**

Cells

LKR-10 is a lung adenocarcinoma cell line derived from K-ras^LA1^ mouse cells [33,34] (kindly provided by Guido Bommer). Human epithelial HeLa cells were from ATCC (ref CCL-2).

Cells were grown in Dulbecco Modified Eagle medium (DMEM, Lonza) containing ultraglutamine and 4.5 gr/L of glucose, and supplemented with 10% of fetal calf serum (Sigma) and 50 units/ml of penicillin/streptomycin (Lonza). Cells were seeded in 24-well plates and were treated with IFN the following day.

Interferons

MuIFN-αA was produced by transient transfection of 293T cells with pcDNA3-IFNαA. Biological activity of the cytokine was determined by cytopathic effect reduction assay. Cells were treated with 100 U/well muIFN-αA or huIFNα (RoferonA, Roche), or with 50 ng/well recombinant muIFN-λ (kindly provided by R. Hartmann, Aarhus university, Denmark) or recombinant huIFN-λ (kindly provided by J.C. Renauld and L. Dumoutier, Univ. of Louvain, Belgium) in a total volume of 500 μl for 24 hours before RNA extraction. This experiment was performed in quadruplicate.

Quantitative RT-PCR

RNA was isolated from cells, reverse-transcribed and subjected to quantitative RT-PCR (RT-qPCR), using SybrGreen and the MyIQ^TM^ apparatus (Biorad). Primer sequences for OASl2 and MxA are described in main text.
